# Light to intermediate oil sheens increase Manx shearwater feather permeability

**DOI:** 10.1098/rsos.220488

**Published:** 2022-10-05

**Authors:** E. Murphy, M. Jessopp, J. Darby

**Affiliations:** ^1^ School of Biological, Earth & Environmental Sciences, University College Cork, Cork, Ireland; ^2^ MaREI Centre, Environmental Research Institute, University College Cork, Cork, Ireland

**Keywords:** chronic oil pollution, oil spills, seabird feather structure, marine conservation

## Abstract

Oil pollution has profound negative impacts on the marine environment, with seabirds particularly vulnerable to oiling, due to the amount of time spent on the sea surface foraging or resting. Exposure to oil can affect feather structure and influence waterproofing, buoyancy and thermoregulation. We investigated the effects of surface crude oil on the feather structure of Manx shearwaters (*Puffinus puffinus*), a seabird species that spends a high proportion of time on the water surface. Sampled body contour feathers were exposed to varying thicknesses of surface crude oil before assessing their resistance to water permeation, increase in mass and clumping of feather barbules. Surface oil as thin as 0.1 µm was enough to increase feather permeability, while greatest impacts on permeability were caused by exposure to dark colour surface sheens 3 µm in thickness. Increases in feather mass of up to 1000% were noted in heavy oiling scenarios due to contact with thicker oil slicks, which may significantly affect wing loading and energetic expenditure.

## 1. Introduction

Industrialization of the marine environment is continuously intensifying, with all marine habitats affected by at least one anthropogenic influence [1]. This places increased pressure on marine ecosystems through stressors including habitat destruction, overfishing and pollution. Oil spills are one form of environmental pollution that poses both immediate and long-term consequences for marine ecosystems. Large-scale oil spill disasters have been frequently recorded over the past number of decades, including the Exxon Valdez (Alaska, 1989) [2], Sea Empress (Wales, 1996) [3], Prestige (Spain, 2002) [4] and Deepwater Horizon (Gulf of Mexico, 2010) [5]. While not as immediately damaging as an acute large-scale leak, persistent or chronic oil pollution can also impact vulnerable marine populations over much greater time scales. The rapid release of large volumes of oil during catastrophic spill events directs immediate focus on rectifying the incident, whereas cases of persistent and chronic oil pollution often occur on a smaller scale but on a much more frequent basis. Such persistent oil spills can also have a greater long-term impact on marine life than rapid spills like Exxon Valdez [6].

The spread of oil from the point of release is a complex process. The volume of oil spilled, along with the chemical composition and stage of refinement can affect the spread of oil across the water surface, where it can be further transported via wind and water currents [7,8]. Crude oils are notorious for spreading rapidly as a thin film coating on the sea's surface. The buoyancy and viscosity of such oils make it a highly dangerous substance that can be problematic for a wide range of marine life, particularly those that interact with the sea surface. Natural breakdown of oils through emulsification in water can occur over time, or through human intervention via the addition of emulsifiers or *in situ* burning. The wider extent of impacts from crude oil spills, however, are not well understood, as slicks can be transported large distances, affecting organisms ranging substantial distances from the original incident [9,10].

One group of marine organisms that are particularly vulnerable to oil pollution is seabirds. This is largely due to their reliance on the marine environment for food, and a high level of interaction with the sea surface for feeding and resting. A comprehensive review identified marine pollution, such as spilled oil, as a global threat to seabird populations [11]. By way of example, the Exxon Valdez and Deepwater Horizon were both probably responsible for over 200,000 seabird deaths [12], though uncertainty around this figure means the true death toll could be much higher [13]. Chronic oil pollution, stemming from sustained accidental or intentional release of oil or oily residues, is a sustained threat to seabirds that receives less attention than catastrophic spills [14].

The location, timing and extent of exposure to oil that seabirds experience directly influence the likelihood of mortality [15], with spills in areas of high seabird diversity and abundance likely to have the greatest impacts on the health and survival of seabird populations. Oiled seabird carcasses washed ashore have often been used to determine mortality rates of seabirds caused by oil pollution at sea [15,16], though this biases mortality estimates toward coastal species that are more likely to wash ashore. Indirect impacts on seabird fitness and reproductive output are more difficult to quantify. Oil contamination can lead to mortality or impact fitness depending on the concentrations of oil encountered. Types of exposure include toxicity following accidental ingestion or inhalation, decreased insulation and a loss of buoyancy [17], and a substantial coating of oil on feathers increasing the overall mass of the bird, requiring it to compensate for this surplus wing loading. This can induce elevated stress, potentially resulting in further physiological issues [14]. Even small volumes of oil have proved problematic for seabirds by affecting the structural integrity of feathers, which influences waterproofing capabilities [17,18]. This has knock-on effects for thermoregulation and energetic expenditure. Although a collection of studies have investigated the concentration or thickness of oil on the water surface required to visibly compromise the functional structure of seabird feathers [19,20], few have explored the effect of oil contamination on the functional waterproofing of feathers through physically testing the waterproofing capabilities of oiled versus non-oiled feathers.

We recognized a gap in knowledge surrounding the severity of oiling required to disrupt feather waterproofing functionality. We hypothesized that any exposure to oil impacts the permeability of feathers. The objectives of this study were to experimentally determine (a) the effect of increasing concentrations of oil on the permeability of feathers by examining the time taken for a set volume of water to penetrate feathers after oiling, (b) the effect of oiling on feather structure and the ability of barbules to interlock and create a waterproof barrier at increasing concentrations of oil, and (c) to quantify the increase in feather mass when exposed to thicker layers of surface oil.

## 2. Material and methods

### 2.1. Sample collection and oiling procedure

Experimentation was carried out on feathers of Manx shearwaters (*Puffinus puffinus*), a pelagic seabird that spends a high proportion of time on the water surface, which leads to an increased vulnerability to oiling [21]. Breast feathers (two or three) from live, breeding Manx shearwaters were plucked under permit in 2014 and 2015 as part of wider tracking studies. Birds were captured at the nest using access tunnels described by Brooke [22] and purse nets at the burrow entrance described by Wischnewski *et al.* [23]. Handling time was kept to a minimum, averaging 7 min per individual, and all capture and sampling procedures were approved by the UCC animal ethics committee and conducted under permits issued by the National Parks and Wildlife Service (C075/2014, 018/2014, C085/2015, 016/2015) and the Irish Health Products Regulatory Authority (AE19130/P004).

Four oil thicknesses corresponding to sheen and slick types observed at sea [24] were used as study treatments (table 1). Intermediate-weight petroleum oils generally spread rapidly over the surface of water from the spill source, initially into a dark black/brown coating of oil known as a slick. With further spreading across the surface, a thin sheen is formed. This is characterized by a silver/grey appearance on the water surface, with an iridescent rainbow evident in direct light. Crude oil was chosen for this study on the basis that it is commonly recognized as a prominent source of chronic oil pollution on the sea surface through leakage from extraction activities, and as it is more viscous than most other processed hydrocarbon fuels, could present a greater risk to species utilizing the water surface near extraction zones [25]. We consider this the most appropriate starting point for examining the influence of oil pollution on feather permeability, where future research may investigate other sources of hydrocarbons found within the same environment. The same crude oil was used for all treatments. We used light to intermediate gravity Oseberg blend crude oil (Gravity = 39.6), extracted from the North Sea and typical of the gravity of crude oils extracted from much of the breeding range of the study species. For this investigation, volume of oil used per treatment was calculated using the standard formula of cylinder volume: (ml or cm^3^) = π*r*^2^*h* (thickness of oil in cm), based on the surface area of the Petri dish used (4.5 cm radius).

**Table 1.** Description of oil treatments used in this investigation. These are based on surface behaviour and appearance of various oil thicknesses described in HAZMAT [22].

treatment	description	oil thickness (µM)	oil volume (µl)
0	control (no oil)	0	0
1	trace colour sheen	0.1	0.64
2	dark colour sheen	3	19.1
3	standard slick	25	159
4	severe slick	75	477

Feather oiling methodology followed similar procedures outlined in O'Hara & Morandin [19]. To prevent the introduction of additional sources of variability, naturally occurring preen oils were not removed, to allow a more accurate representation of the natural state of the feather structure. First, 20 ml of seawater at room temperature were placed in a clean Petri dish using a graduated cylinder. One dish with an associated oil treatment was prepared at a time to prevent evaporation that could impact viscosity and general oil composition. Crude oil treatments were applied to the seawater surface using micro pipettes. Treatments 1 and 2 generally dispersed with ease, and for treatments 3 and 4, the tip of the pipette was used to gently distribute the oil across the entire surface of the water to obtain a more even coverage.

Feathers for experimentation were chosen based on an even spread of barbs, i.e. equal distance between each barb, and no obvious holes or clumping prior to treatment that may have occurred during sampling and storage. Each feather was gently blown using a light, cool setting of a hairdryer for 5 s to encourage a more even spread of barbs along the feather's rachis and were weighed using a high sensitivity electric weight balance (± 0.0001 g). Using tweezers, the feather was held by the calamus and placed convex side down on the surface of the treatment in the Petri dish. Feathers were left stationary for 15 s, then using the tweezers were swiped three times over the surface of the treatment, and then left stationary on the surface for a further 15 s. Feathers were weighed again, and the length of the feather from the start point of the barbs along the rachis to the tip of the barbs was measured. Feathers intended for percolation testing were left to sit for 1 min following oiling before being tested, and freshly oiled feathers for digital microscopy were prepared 24 h in advance, where samples were stored convex side up on paper towel in a sealed container. This was to allow for gradual draining of surplus oil from the feather's surface to improve the visibility of barbule interactions of heavily oiled samples.

### 2.2. Percolation testing and amalgamation indices

**Figure 1.**
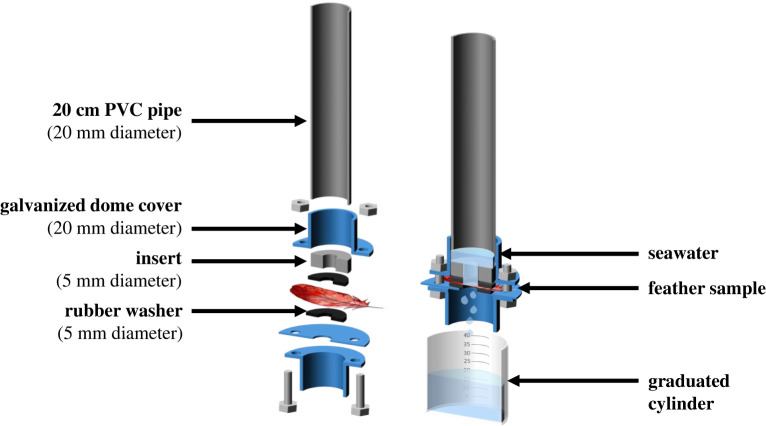
Diagram of laboratory set-up for percolation testing. Displayed are disassembled (left) and assembled percolation tester, showing percolation of 25ml of seawater (right). To run the percolation test, the treated feather must be clamped between the two rubber washers, visible on the diagram on the left, and water let percolate from the top of the apparatus to the graduated cylinder below.

Percolation tests were used to record the time taken for water to penetrate through feathers following different degrees of oil exposure to determine the impact of oiling on the waterproofing quality of feathers. Feathers (*n* = 180) were placed oiled (convex) side facing up, held within a rubber washer with a hole 5 mm in diameter exposing a section within the central third of the feather's surface, avoiding any obvious gaping/clumping (figure 1). The washer was held between two plates with an opening on the surface with a 20 cm PVC pipe attached. The plate beneath was situated over a graduated cylinder. Twenty-five millilitres of seawater were poured into the PVC pipe on top of the feather, and a timer was simultaneously started. The time taken for 20 ml to percolate through the feather was recorded by observing the volume of water dropping into the graduated cylinder below. Following tests, feathers were stored in labelled containers for microscopy.

The barbule amalgamation index (AI), based on procedures outlined by O'Hara & Morandin [19], was used to identify the degree of clumping resulting from exposure to oiling treatments. Samples used for percolation testing (*n* = 10), as well as freshly oiled samples (*n* = 10) were examined microscopically, examining two feathers per treatment from each preparation type. For feathers that underwent percolation testing, the 5 mm diameter section water had passed through was examined to investigate feather structure following oiling and water throughput. Freshly oiled samples were used to examine the direct effect of oil on feather microstructure prior to exposure to water. To ensure the feather lay flat when examining under the microscope, the upper and lower thirds were cut off, leaving only the central section for analysis. A Leica VZ700C digital microscope was used to assess four areas of the barbules of each feather, two either side of the rachis, or within the area exposed to water percolation. Feathers were examined under the highest magnification lens (approx. 2500×) and multiple images (20 to 50) at varying focal lengths were compiled to create stacked images to increase the depth of field and improve the quality of the images recorded. A total of 16 images were therefore recorded at each treatment level, with 8 images recorded for each of the two outlined preparation types. AI were recorded for each feather from each treatment by calculating the mean spread/clumping of barbules across a length of 21 to 25 barbules. A barbule at the centre of the barb was chosen as a starting point and 10–12 barbules were counted outward from either side of this central point. Barbules that were not in contact/clumped with others were given a value of 1, and any adjacent barbules clumped together were valued with the number of barbules within the clump. The AI was determined by calculating the mean value for each feather, indicating the mean number of barbs per clump. A value of 1 is therefore the minimum, as this signifies a mean of 1 barbule per clump, and none of the barbules had adhered to each other. The higher the AI, the more barbules were clumped together.

### 2.3. Data analysis

Data analysis was performed using R v. 4.0.4 (www.R-project.org/). The *tidyverse* (cran.r-project.org/web/packages/tidyverse/), *DescTools* (cran.r-project.org/web/packages/DescTools/) and *ggplot2* (cran.r-project.org/web/packages/ggplot2/) packages were also used. A Shapiro–Wilk test was performed on the mass of feathers pre and post oiling to determine the normality of these data, with subsequent non-parametric Wilcoxon-signed rank testing used to test for differences in mass of feathers before and after applying oil treatments. The percentage change in mass due to oiling was also calculated for each feather sample and tested using linear modelling to examine the relationship between percentage change in mass and oil treatment. A standardized size was determined for each feather, calculated by dividing the feather mass before treatment by the feather length. Generalized linear models (GLM) with a Gaussian error structure and identity link function were used to relate percolation time to oiling treatment, initially using mass prior to oiling treatment and feather size as covariates. Highly collinear covariates were identified by calculating variance inflation factors (VIFs) and were removed. Akaike's information criterion (AIC) values were used to select the best fitting model structure. The final GLM was fitted using the retained covariates. The effect of oiling on AI was tested using analysis of variance (ANOVA), with AI as the response variable; oil treatment, preparation type, and their interaction were included as explanatory variables.

## 3. Results

### 3.1. Feather mass and size

**Figure 2.**
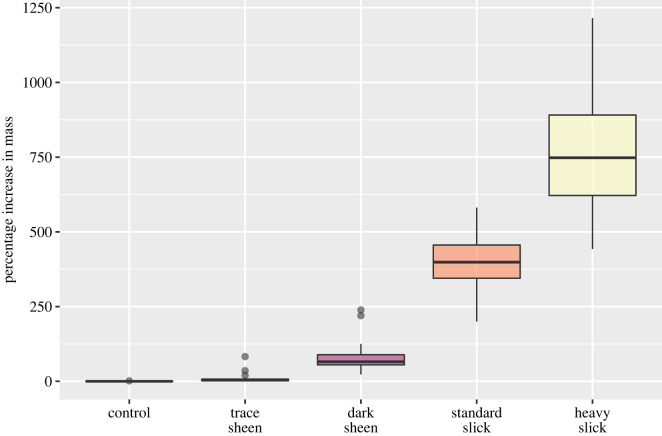
The percentage weight gain of feathers for each treatment type (as described in table 1).

Feathers were significantly heavier following oiling for all treatment levels (Wilcoxon-signed rank test, all *p* < 0.001). The concentration of oil in each treatment explained 90% of the variation in change in mass observed in feathers before and after treatment (linear model, *F*_4,150_ = 337.3, *p* < 0.01, figure 2). Mean percentage increase in mass was proportionate to the oiling treatment. Light sheens, dark sheens and standard slicks led to 8%, 78% and 399% mean increase in mass, respectively. In the heavy slick treatment, feather mass increased by over 1000% in some samples (mean 749%).

### 3.2. Percolation testing

**Figure 3.**
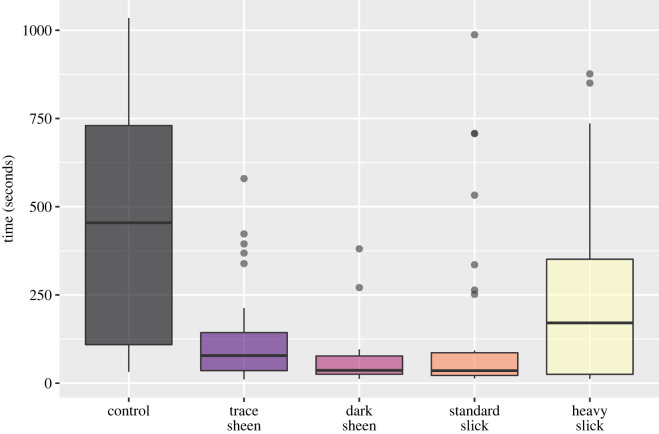
Time required for 20 ml of seawater to percolate through feathers treated with various thicknesses of surface oil. Treatments are described in table 1.

Figure 3 shows the time taken for 20 ml of seawater to percolate through each feather across oil treatments. Data are widely spread for control and heavy oil treatments while the dark colour sheen treatment represented the lowest average time required for percolation (median = 36.3 s).

Variance inflation factors (VIFs) identified high collinearity between pre-treatment mass, post-treatment mass and standardized feather size. Variables with lowest VIFs (pre-treatment mass and feather size, VIF < 2) were identified as not being collinear with oiling treatment and so were used in separate initial models for selection using AIC. The best fitting model was identified as that with the lowest AIC value (AIC = 1996.3), which retained treatment alone as an appropriate descriptor of percolation time. Model checks showed residuals followed a normal distribution. All oil treatments led to a significantly lower percolation time than the control. Sheens had a greater impact on percolation time than slicks. Feathers exposed to severe slicks were least impacted in terms of percolation time (table 2).

**Table 2.** Result of the generalized linear model (GLM) comparing percolation times as a response to oiling treatment. *p*-values of less than 0.05 are taken as significant (italic values).

term	estimate	s.e.	*t-*value	*p-*value
(intercept)	452.7	42.0	10.8	*<0.001*
trace sheen	−323.5	59.4	−5.4	*<0.001*
dark sheen	−388.8	59.4	−6.5	*<0.001*
standard slick	−300.0	59.4	−5.1	*<0.001*
severe slick	−212.2	59.4	−3.6	*<0.001*

### 3.3. Amalgamation index

**Figure 4.**
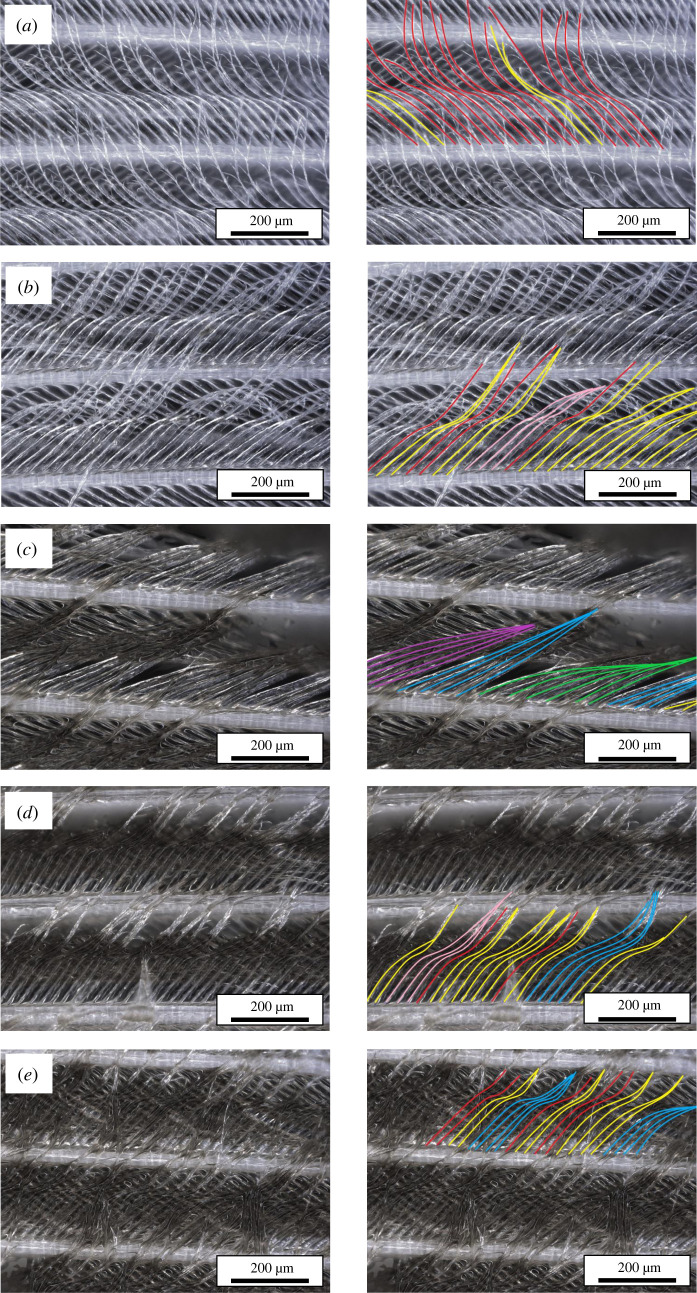
Amalgamation annotation of Manx shearwater feathers. Left: the distribution of barbules along the barb at increasing levels of oil treatment. Oil treatments are (*a*) control, (*b*) trace colour sheen – 0.1 µm, (*c*) dark colour sheen – 3 µm, (*d*) standard slick – 25 µm, and (*e*) severe slick – 75 µm, (table 1). Right: amalgamation Index analysis has been marked to display spread/clumping reflected in mean AI calculations, with the following colours representing values determined for clump sizes: Red = 1, Yellow = 2, Pink = 3, Blue = 4, Purple = 5, Green = 6.

**Figure 5.**
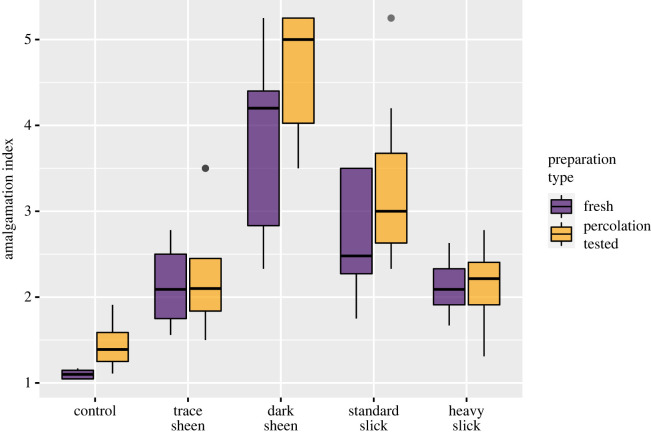
Amalgamation indices per treatment. Results are separated by percolation-tested (yellow) and freshly prepared (purple) feather samples (*n* = 80). Amalgamation index was calculated based on procedures established by O'Hara & Morandin [19].

All oil treatments showed increased clumping compared with control feathers, with the greatest degree of clumping of barbules in feathers exposed to a dark colour sheen (intermediate oiling, figures 4 and 5). Both oil treatment and preparation type (freshly oiled versus oiled and percolation tested) had a significant effect on AI (ANOVA, *F*_4,75_ = 40.69, *p* < 0.05); however, there was no significant interaction between treatment and preparation type, indicating a consistent effect of oiling on AI regardless of whether water was subsequently passed through the feathers. However, there is a significant difference between the AI of percolation-tested and fresh feathers in the control category. Percolation testing led to a higher AI than in feathers that were not percolation tested prior to examination, meaning water throughput also has a clumping effect on seabird feather structure regardless of the introduction of oil. This clumping effect is less pronounced than that recorded under any of the oiling scenarios. *Post hoc* comparisons showed significant differences in AI between all oil treatments and the control as well as significant differences between all treatments, except between trace colour sheen and severe slick treatments (1 and 4).

## 4. Discussion

This study describes a clear effect of oiling on the water permeability of feathers, and a relationship between the level of oiling and associated effects on feather mass, permeability and structure. Interestingly, the greatest effect on feather permeability was from intermediate levels of oiling, which also provided the highest AI. This suggests that thin oil sheens consistent with well-dispersed spills have a greater effect on feather permeability than thick oil treatments, but this must be viewed in the context of other impacts from heavier oiling scenarios, such as toxicity from ingestion or absorption and increase in body mass. Water repellence is facilitated by a consistent feather structure, where any interference can have negative implications for waterproofing. Within a feather's typical structure, an air–water interface is produced across the rough, porous surface, which ensures the prevention of water penetration. Introducing oil contaminants interrupts the feather's structural integrity by preventing barbules from interlocking, resulting in gaps between feather barbs [26,27].

Intermediate sheens that severely compromise feather structure, consistent with chronic oil pollution conditions in the marine environment, are particularly hazardous to seabirds, as the thin layer of oil rests on the sea surface, where many seabirds spend time resting and foraging [28,29]. Such chronic oil pollution has been determined to cause greater seabird mortality compared with larger but more infrequent spills [30], although accurate estimations of seabird mortality related to chronic oiling have proved difficult to determine. Results from this study, and others like it, can provide some understanding surrounding smaller but more prevalent and damaging volumes of oil on the sea's surface [31]. It was hypothesized that larger volumes of oil exposure would result in greater impacts on feathers' functional structure. This study, however, shows that even small amounts of oil can disrupt the feather's barrier against water infiltration. Under oil slick conditions, surface tension is created between the oil and the water, where barbs and barbules remain more evenly spread through the balance of adhesion and repulsion forces between adjacent barbs created by the heavy layer of oil, and more pressure is required for water to permeate through the barbs. This is similarly reflected in seabird species whose feathers can appear wet following contact with water, where the repulsion and adhesion forces created by the water trapped within the feather microstructure prevents further penetration of water down to layers closer to the body [27]. In heavily oiled samples, the barbules are held in a more uniform distribution by the forces created from the heavy layer of oil, which is described by a low AI. For an intermediate level of oiling, only some barbules are held together in clumps, but not enough oil is present to completely coat the feather surface, allowing water to percolate through the gaps between the clusters of barbules.

Natural oils play an important role in improving hydrophobicity in seabird feathers. It has been suggested that preen oils, rather than directly determining hydrophobicity, encourage an even arrangement of barbs and barbules that in turn enables the waterproofing function of feathers [32]. The weight of such a light coating of oil applied during preening does not impact the individual but does, however, provide the optimal level of oil for waterproofing. The structural properties probably differ between natural-based preen oils, composed of predominantly fatty acids and alcohol moieties, and the volatile liquid hydrocarbons of petroleum-based oils, with different compositions and viscosities fundamentally impacting the feathers' structural integrity [33,34]. Some clumping was recorded following water throughput in feathers not exposed to crude oil, though the extent of this was highly reduced in comparison with feathers that were exposed to oiling treatments. Water throughput is therefore likely to affect feather structure even when feathers are not contaminated, though evidently far less than when they are, and may be more easily corrected by preening and preen oils.

All feathers exposed to crude oil became significantly heavier, and unsurprisingly, the percentage change in mass increased in proportion to the concentration of oil per treatment. The mean percentage increase in mass of feathers was 400% and 750% when exposed to standard and thick oil slicks respectively, with a maximum increase of 1215% in the severe slick treatment. With such a large increase in mass recorded for one feather alone, the cumulative effect of oil contamination on a large proportion of one individual's feathers may substantially increase wing loading. The degree of oiling required for wing loading, locomotion and buoyancy to be impacted are difficult to quantify, as this will also depend on the number and location of feathers affected.

Maggini *et al.* [35] recorded substantial effects of even light crude oil coating on tail, wing and breast feathers of western sandpipers (*Calidris mauri*), a migratory shorebird. Effects were predominantly mechanical, impairing flight capabilities, which in turn negatively impacts migration efficiency, foraging, chick provisioning and predator evasion through ‘parasite drag’. Physiological effects of oiling have also been suggested, with water penetration strongly relating to heat loss in seabirds. Hypothermia and increased feather mass can cause elevated metabolic rate, and if not compensated for through increased food intake, can cause decreased muscle and fat content and an overall deterioration of the fitness of the individual. Species such as Cassin's auklets (*Ptychoramphus aleuticus*) and double-crested cormorants (*Phalacrocorax auratus*) have partially wettable plumage, meaning they are capable of compensating for some degree of heat loss [27,36]. This does, however, come with a cost, where a proportion of time that could be spent foraging must be sacrificed to allow for time for drying off. Manx shearwaters, and other small diving species, are more susceptible to effects of decreased insulation, where thermal conductance increases with water penetration, caused by severe clumping of feather barbules or heavy oil coverage causing heat to escape easier through the liquid layer compared with an insulative layer of air [27,37]. Determining the extent of oil penetration from water being forced through multiple layers may present further insight into the implications for feather structural integrity and waterproofing. The non-significant interaction between oil treatment and preparation type implies that clumping of barbules at different concentrations of oil coverage does not vary depending on percolation. However, as waterproofing is impaired when feathers are exposed to oil, degradation of the feathers' condition is probably exacerbated with subsequent water throughput when compared with non-oiled feathers, to a greater degree than is explained by this interaction.

Seabird behaviour can influence the degree of impact on a species when exposed to oil on the surface of water. Manx shearwaters are particularly susceptible to the negative impacts of oil pollution, given that they interact with the water surface for protracted periods of time, increasing their risk of interaction with surface contaminants [38]. This species also spends a substantial amount of time at depth while feeding, where pressure will accelerate percolation of water through oiled feathers [39]. Oiled individuals who are regularly diving may gradually remove a substantial amount of oil coating from feathers when submerged in seawater [40], though we have shown that even minute amounts of oil can jeopardize the waterproofing of feathers. This suggests that the negative effects of oiling on feather waterproofing may be sustained over protracted time periods and may be compounded by increased body mass and toxic oil ingestion due to preening [41].

There is probably a large variation of preen oil concentrations naturally present on feathers pre-anthropogenic oiling, which may account for the high variance in the percolation times in control feathers observed in this study. It would, however, be inappropriate to remove preen oils prior to experimentation as this is likely to introduce new sources of variability, as well as becoming a poorer resemblance of a natural state. The occurrence of preening behaviour may also counteract the impacts of oiling to an extent in natural scenarios. Determining the extent to which seabirds may remove contaminants from their feathers and the speed at which this behaviour occurs would be difficult to replicate in a laboratory environment, without the involvement of live individuals. Not accounting for the removal of oil via preening may overestimate the effect size, particularly with respect to increased mass following oil treatment. However, preening may also exacerbate the negative impacts of oiling, where oil may be forced further into the layers of the birds’ feathers, or toxicity following accidental ingestion [41]. Petroleum oil ingestion has been linked to reduced reproductive success, including egg viability and hatching success [42], though this may be caused in part by increased time spent preening feathers rather than spent foraging following oil exposure [18,41].

With seabird populations declining globally, exacerbated by the influence of anthropogenic pollution, it is vital to understand the extent of such impacts and how efforts can be made to counteract them. Manx shearwaters are a particularly susceptible species given their increased risk of interaction with surface contaminants during extended periods spent on the water surface and their deep dives exposing them to high pressures that may force water through damaged feathers. Our study suggests that even light levels of oiling can have serious negative effects on vulnerable seabirds, highlighting the need for further efforts to reduce even minor spills and improve standards surrounding relevant infrastructure in the marine environment.

## Data Availability

Data and relevant code for this research work are stored in GitHub: https://github.com/JamieHDarby/feather_oiling, and have been archived within the Zenodo repository: https://doi.org/10.5281/zenodo.7078889 [43].
